# Carboxymethyl chitosan reduces inflammation and promotes osteogenesis in a rabbit knee replacement model

**DOI:** 10.1186/s12891-020-03803-3

**Published:** 2020-11-24

**Authors:** Feng Liu, Hai-Yan Li, Zhen Wang, Hai-Ning Zhang, Ying-Zhen Wang, Hao Xu

**Affiliations:** 1 Department of Joint Surgery, The Affiliated Hospital of Qingdao University, Qingdao, 266000 Shandong China; 2 Wuhan Fourth Hospital; Puai Hospital, Tongji Medical College, Huazhong University of Science and Technology, Wuhan, Hubei China; 3 Center for Musculoskeletal Surgery, Charité University Medicine Berlin, Berlin, Germany

**Keywords:** Carboxymethyl chitosan, Knee arthroplasty, Inflammatory, Osteogenesis, Rabbit model

## Abstract

**Background:**

The major causes of failure after total knee arthroplasty (TKA) include prosthesis loosening and infection. This study aimed to investigate the role of carboxymethyl chitosan (CMC) in knee arthroplasty.

**Methods:**

A total of 20 New Zealand white rabbits that were divided into two groups (10 in the control group and 10 in the chitosan group) were included in the study. They underwent TKA surgery, and all were implanted with titanium rod prostheses; the prosthesis in the chitosan group was coated with CMC. After 12 weeks, all rabbits were euthanized, and the following analyses of some specific surface membrane tissues around the prosthesis were performed: X-ray analysis; micro-computed tomography scan; haematoxylin and eosin, Van Gieson, and Von Kossa staining; reverse transcription polymerase chain reaction; and Western Blotting.

**Results:**

The result of CCK8 test showed CMC can promote cell proliferation and increase cell viability. Radiological result showed better amount of bone deposits and more bone formation in the chitosan group. HE staining result showed CMC reduces inflammation around the prosthesis. The VG and Von Kossa staining results showed CMC can promote bone deposition around prosthesis. And according to the results of PCR and WB, the OCN content was higher in the chitosan group, while the MMPs content was lower. The chitosan group has an increased OPG/RANKL ratio than the control group.

**Conclusion:**

CMC can effectively inhibit the inflammatory response around the prosthesis and osteoclast activation and promote osteogenesis by interfering with the osteoprotegerin/receptor activator of nuclear factor kappa-Β ligand/receptor activator of nuclear factor kappa-Β signalling pathway.

## Background

Artificial knee arthroplasty is an effective method for treating end-stage knee joint disease. Annually, millions of patients undergo this operation worldwide. Most patients have reconstructed joint function, which improves their quality of life [1]. The number of patients undergoing total knee arthroplasty (TKA) is increasing, and revision surgeries are also consistently developing [2]. However, complications including pain and dysfunction are inevitable. Revision surgery is relatively expensive, increasing the patients’ medical burden. Moreover, revision surgery has less satisfactory results than the first TKA [3]. Therefore, decreasing the revision rate after the first TKA has become one of the most interesting topics in the field of joint surgery.

A current study shows that the most common causes of revision after the first TKA are infection and aseptic loosening [4]. Periprosthetic infections often occur in the early revision phase within 2 years [5]. The most common pathogen of infection is *Staphylococcus aureus*, which indicates that the main infection originates from the patient’s skin or the surgical field and improper disinfection of the equipment [6]. Aseptic loosening of the prosthesis is significantly common years later in late revision phase 2 [7]. The reasons for aseptic loosening in the early and late stages are poor bone growth and bone absorption, respectively. At present, apart from good techniques and continuous optimization of prosthesis materials, there is no optimal method for preventing failure after the first TKA. Infection can be primarily prevented by administering prophylactic antibiotics. Based on research involving a rabbit *Staphylococcus* animal model, Gosheger et al. proposed that a silver-coated artificial joint can reduce the incidence of postoperative infection [8]. The prevention of aseptic loosening of the prosthesis is mainly to inhibit the inflammatory response [9] and interfer with the osteoprotegerin (OPG)/receptor activator of nuclear factor kappa-Β ligand (RANKL)/receptor activator of nuclear factor kappa-Β (RANK) pathway, which promotes bone growth, reduces osteolysis by inhibiting the function of osteoclasts [10]. Therefore, if a material can prevent infection, and enhance the contact between the bone tissue and prosthesis, which is very important for the success of joint replacement.

Chitosan is a high-molecular-weight polymer produced after deacetylation from keratin [11]. Keratin is widely found in the cell walls of fungi and shells of crustaceans and insects. Keratin molecules form chitosan through N-terminal deacetylation [12]. Chitosan’s hydrophilic surface can also promote cell adhesion, proliferation, and differentiation. Carboxymethyl chitosan (CMC) has recently been introduced. CMC not only retains the excellent properties of chitosan but also has stronger water solubility and biological activity [13]. Chitosan and its derivatives have antibacterial activities against various microorganisms, and its broad-spectrum antibacterial activity has been confirmed by many studies [14, 15]. The antioxidant effect of CMC is related to the hydroxyl and carboxyl groups in the polymer chain. They can easily absorb hydrogen atoms in free radicals to form stable polymer groups [16].

Despite the abovementioned characteristics of chitosan, studies on the use of chitosan in TKA have not yet been conducted. We established a cementless TKA model to simulate the early reaction stage of joint prosthesis implantation with the use of chitosan to investigate its effect on the prosthesis surroundings.

## Methods

### Animals and materials

1. A total of 20 adult New Zealand rabbits were randomly divided into the chitosan group and the control group, with 10 rabbits in each group. The rabbits were purchased from the Hubei Provincial Center for Disease Control and Prevention (Wuhan, Hubei, China). The average weight of the rabbits was 2.83 kg. All rabbits were housed in cages with a constant temperature of 20–25 °C under a 12/12-h light-dark cycle. Food and water were accessed freely. All the animal procedures were following the revised Animals (Scientific Procedures) Act 1986 in the UK and Directive 2010/63/EU in Europe, and were approved by the Ethics Committee of Wuhan Fourth Hospital, and conducted following the relevant guidelines and regulations (Approval Number: 20191947).
2. Titanium rod prostheses purchased from Stryker Co. Ltd., USA, were cylindrical in shape (length, 2 cm; diameter, 0.5 cm) and sterilized using a high-pressure steam before use.
3. Chitosan hydrogel was purchased from Shijiazhuang Yishengtang Medical Product Co. Ltd. in China. CMC was used as the main component and was configured with a physiological equilibrium solution with a concentration of 25 mg/ml. It was a colourless transparent viscous liquid that was stored in a sterile area and sealed in a 4 °C refrigerator for single use. We diluted it according to the results of the CCK8 test and selected the concentration with the best cell proliferation ability for the experiment.
4. Wuhan Fourth Hospital provided animal cages and an experimental operation platform.

### Detection of the value-added ability of carboxymethyl chitosan

RAW264.7 cells that were in the logarithmic growth phase were used for the experiments. The cell density was adjusted to 5 × 10^3^/ml with DMEM. A total of 0 (control group), 0.1, 0.2, 0.5, 1, 5, or 25 mg/mL carboxymethyl chitosan was added to cells in each well of a 96-well plate, and the plate was placed in an incubator for 24 h. A total of 10 μL of cck8 reagent was added to each well, and the plate was incubated at 37 °C with 5% CO2 for 4 h. After incubation, the solution was mixed thoroughly, and the absorbance at 450 nm was measured with a microplate reader. Cell proliferation rate = absorbance value of experimental group-absorbance value of blank group / absorbance value of the control group-absorbance value of the blank group. The concentration with the least effect on cell activity was chosen for subsequent experiments.

### Animal surgery

**Fig. 1:**
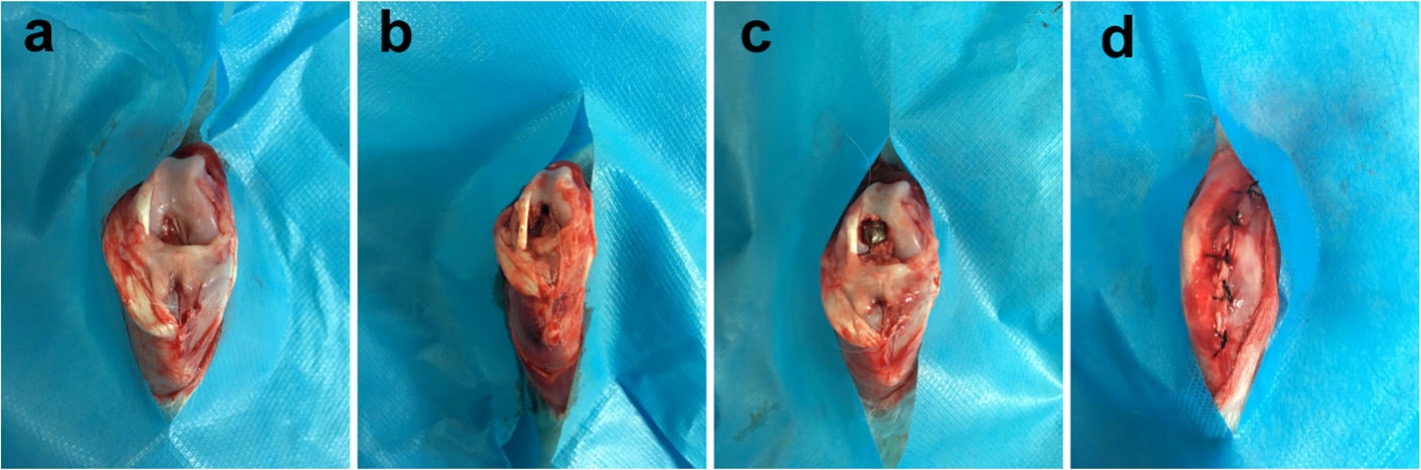
Operative process of titanium rod prosthesis implantation surgery(*n* = 20). **a** The distal femur of the knee prosthesis was exposed. **b** We opened and expanded the femoral medullary cavity with drills of increasing size. **c** The titanium rod prosthesis was implanted into the femoral medullary cavity. **d** The incision was closed after thorough rinsing

All rabbits were adaptively fed for 1 week before surgery. The animals were anaesthetized intramuscularly with xylazine hydrochloride 0.2 ml/kg according to our previous study to ensure that the animals were completely unconscious and painless during the operation [17]. The animal is considered to be in a fully anesthetized state when breathing is regular and stable, the corneal reflex is slow, the whole-body muscles are relaxed, and there is no response when pinching the skin. The rabbit was then placed in a supine position on an operating table. The right knee was used as the surgical side. The sterile towel covered the surgical area, and the medial skin incision and paracondylar approach were used to expose the articular cartilage along the subcutaneous tissue and the joint capsule layer. A hole was drilled above the endpoint of the posterior cruciate ligament in the femoral intercondylar fossa to open the medullary cavity, and the medulla was expanded from 0.3 cm to 0.5 cm along the longitudinal axis of the femur with a depth greater than 2 cm. The titanium rod of the chitosan group was immersed in the CMC solution for half an hour and subsequently implanted in the femoral bone marrow cavity. Each layer of tissue was closed after the medullary cavity was flushed with saline (Fig. 1).

### Postoperative management

The principle of aseptic operation was followed, and 400,000 units of intramuscular injection of penicillin was administered twice a day for the first 3 days after operation. None of the animals had any lower extremity fixation.

### Animal sacrifice and tissue harvest

All animals were euthanized with pentobarbital sodium of 200 mg/kg through the ear vein injection to achieve painless euthanasia of all rabbits after 12 weeks. Synovial tissue on the ipsilateral side of the knee joint was extracted and placed in 10% zinc formalin to prepare paraffin sections. The titanium rod segment with the right distal femur was collected, and the bone was cut off using a hacksaw 2 cm away from the knee joint surface. The distal part was stored frozen at − 80 °C, and hard tissue sections were prepared. The residual boundary membrane tissue around the two titanium rods was retained; one part was placed in ribonucleic acid (RNA) lysing solution which sealed and stored in liquid nitrogen, and the other part was placed in a 1.5-ml test tube which stored in − 80 °C refrigerators. Both were srored for reverse transcription polymerase chain reaction (RT-PCR) and western blotting (WB).

### Radiological evaluation

The X-ray film of the right knee joint was irradiated to observe bone formation. Micro-computed tomography (CT) was performed to reconstruct the osteogenesis around the titanium rod, and bone tissue volume/total volume of bone tissue and prosthesis (BV/TV) was calculated [18].

### Bone and synovial tissue staining and analysis

The synovial tissue of the knee joint was treated with paraffin sections and stained with haematoxylin and eosin (HE). Bone containing titanium rods was processed by non-decalcified hard tissue sections. The non-decalcified bone tissue retained the structure of the trabecular bone and accurately reflected bone growth, which was convenient for the study of bone growth and absorption. Bone tissue was cut into 50-μm sheet structures and cut along the long axis of the femur using a thin slicer. The hard sections were subjected to Van Gieson (VG) and Von Kossa staining. Based on the VG staining, the bone-prosthesis contact rate(B-PCR, the length of the circumference of the bone in contact with the prosthesis/total circumference of the prosthesis [19]) and bone volume percentage (BVP, squares of the bone tissue in a1 mm area around the prosthesis/total squares of the 1mmarea around the prosthesis [20]) were analysed. Von Kossa staining can be used to measure the number and area of calcium salt deposit islands (CSDIs) around the prosthesis [21].

### Western blotting

The tissue stored in liquid nitrogen was moved to an Eppendorf tube, and lysis buffer was added to lyse the cells. The protein concentration was quantified using a bicinchoninic acid (BCA) protein concentration determination kit. A 12% sodium dodecyl sulfate polyacrylamide gel electrophoresis (SDS-PAGE) gel was prepared, the protein sample was loaded, electrophoresis was performed, the proteins were transferred to a membrane, and the membrane was blocked and incubated with the indicated antibodies, namely, mouse monoclonal antibody matrix metalloproteinase-9 precursor (MMP9,Abcam,Ab58803) and osteocalcin (OCN,Abcam,Ab13420), rabbit polyclonal antibody OPG (Bioss,Bs-20624R) and RANKL (Bioss,Bs-2064R), and rabbit polyclonal β-actin, at 4 °C. After 24 h, membranes were rinsed and subsequently incubated with horseradish peroxidase (HRP)-labelled goat anti-mouse (Wuhan Boster Biological Technology.,LTD,BA1051) and goat anti-rabbit (Wuhan Boster Biological Technology.,LTD,BA1054) secondary antibodies for 2 h at room temperature. Membranes were developed in a dark room with the electrochemiluminescence (ECL) light-emitting kit. The Bio-Rad Gel Imaging System was used to collect the images and to perform greyscale scanning analysis of the images.

### Real-time polymerase chain reaction

Total RNA was extracted using TRIzol reagent, and 2 μg of total RNA was used for the reverse transcription reaction. The reverse transcription conditions were as follows: 25 °C for 5 min, 50 °C for 15 min, 85 °C for 5 min, and 4 °C for 10 min. Two microlitres of the above reaction solution was used for PCR, and the following PCR conditions were used: 50 °C for 2 min, 95 °C for 10 min, 95 °C for 30 s, and 60 °C for 30 s, with a total of 40 cycles. The primers were designed using Primer Premier 5.0 software and verified by Blast. The primer sequences are listed in Table 1. Five microlitres of the amplified product was used for analysis by 1% agarose gel electrophoresis, pictures were taken under an ultraviolet light, and the greyscale value was determined using ImageJ 1.45 s software. The relative expression level of the target messenger RNA (mRNA) is expressed as the ratio of the greyscale values of the target band and the internal reference, namely, glyceraldehyde 3-phosphate dehydrogenase.

**Table 1:** Primer sequence

Gene	Upstream Primer	Downstream Primer
MMP9	GTACTCGACCTGTACCAGCG	TTCAGGGCGAGGACCATAGA
OCN	AGCAAAGGTGCAGCCTTTGT	GCGCCTGGGTCTCTTCACT
OPG	GGAACCCCAGAGCGAAATACA	CCTGAAGAATGCCTCCTCACA
RANKL	CAGAAGATGGCACTCACTGCA	CACCATCGCTTTCTCTGCTCT
GAPDH	ATGGGGAAGGTGAAGGTCG	GGGGTCATTGATGGCAACAATA

### Statistical analysis

Image-Pro Plus (IPP) 6.0 was used for graphics and data processing. Data analysis was performed using Graph Pad Prism 6.03 data results for standard deviation analysis. The data model used one-way analysis of variance, with a *p* value of 0.05 considered statistically significant.

## Results

### CCK8 test

**Fig. 2:**
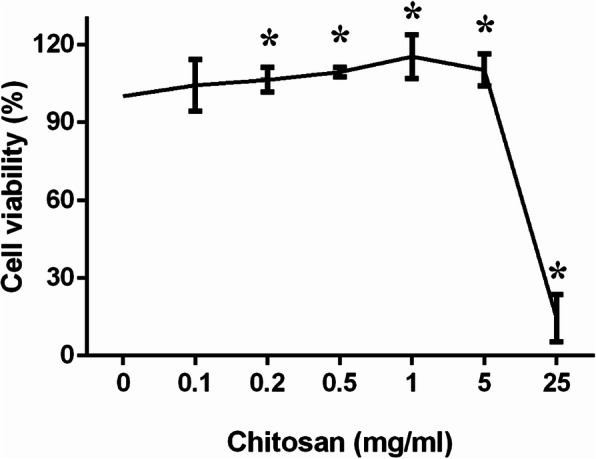
Chitosan at a concentration of less than 5 mg/ml did not affect cell viability. *: *p* < 0.05 compared with control

CMC can promote cell proliferation and increase cell viability. In the range of 0–1 mg/ml, as the concentration of CMC increased, the cell proliferation rate increased. At 25 mg/ml, CMC inhibited cell proliferation and had obvious cytotoxicity. We chose 1 mg/ml for subsequent experiments (Fig. 2).

### Radiological osteogenesis

**Fig. 3:**
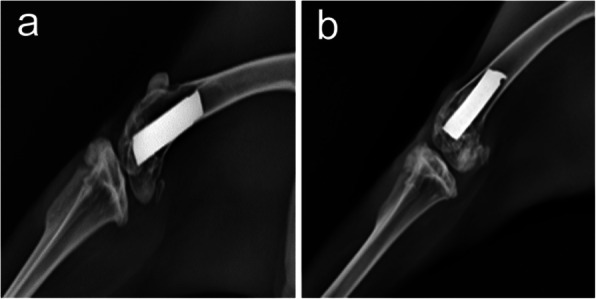
Lateral X-ray of the right femur. **a** Osteoporosis around the prosthesis in the control group(*n* = 10) (**b**) Small amount of bone deposit in front of prosthesis in the chitosan group(*n* = 10)

**Fig. 4:**
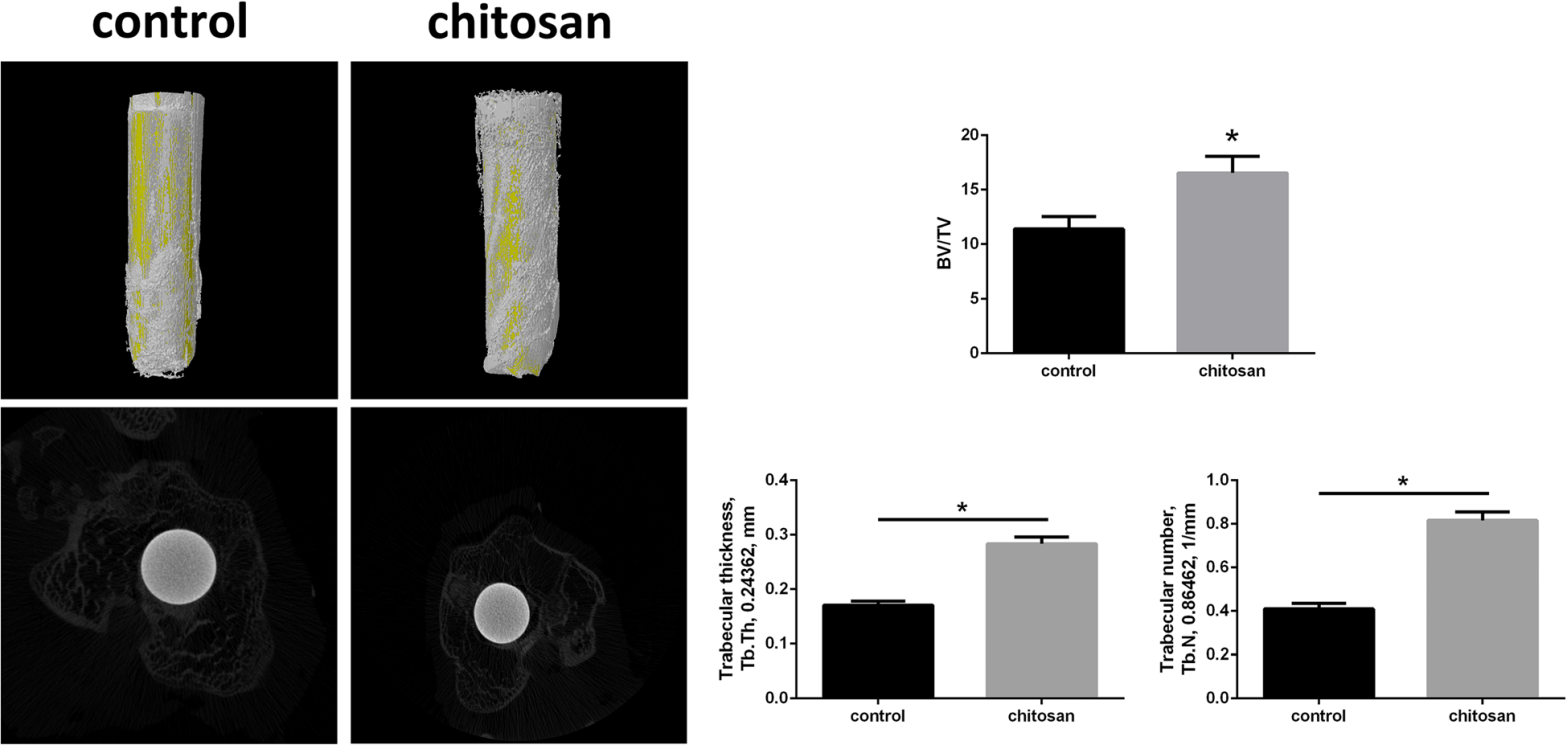
Micro-computed tomography three-dimensional reconstruction and cross section of osteogenesis around the prosthesis. **a** Bone deposition scattered around the prosthesis in the control group(*n* = 10). **b** Bone deposition on the surface of the prosthesis in the chitosan group(*n* = 10). **c** Comparison between the two groups, bone tissue volume/total volume, Trabecular thickness and number were statistically significant (*p* < 0.05)

After 12 weeks of feeding, two groups of animals were irradiated with the right knee joints. The radiolucent area is wider than 2 mm around the prosthesis in the control group. Compared with the control group, the chitosan group had more obvious bone islands deposition around the prosthesis (Fig. 3). Three-dimensional reconstruction of micro-CT showed that there was significantly more bone formation around the prosthesis in the chitosan group than in the control group(*P*<0.05). The mean BV/TV value of the 10 samples in chitosan group was 16.547 ± 1.523SD, and of the 10 samples in control group was 11.384 ± 1.139SD. The cross-sectional micro-CT scans showed that there were trabecular bone growth around the two groups of prostheses. The mean trabecular thickness value of the 10 samples in chitosan group was 0.2837 ± 0.01210SD, and of the 10 samples in control group was 0.1709 ± 0.01210SD. Mean value of trabecular number in chitosan group was 0.8158 ± 0.03838SD, and in control group was 0.4098 ± 0.02562SD.The data showed that the thickness and number of trabecular bone in the chitosan group were higher than those in the control group, and the difference was statistically significant (*P* < 0.05) (Fig. 4).

### CMC reduces inflammation

**Fig. 5:**
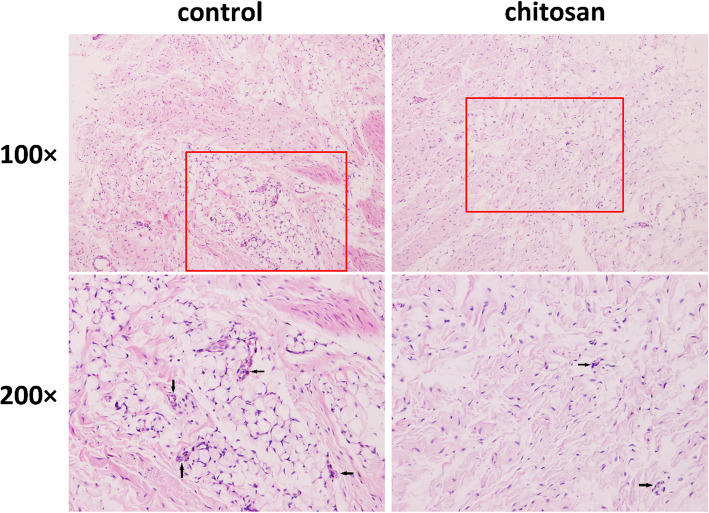
Haematoxylin and eosin staining of the synovial membrane of the knee joint (100x, bar = 100 μm; 200x, bar = 50 μm). Black arrows indicate inflammatory cells. **a** A large amount of inflammatory cells infiltration was observed in the control group(*n* = 10). **b** A small amount of Inflammatory cells infiltration was observed in the chitosan group(*n* = 10)

HE staining showed a significant inflammatory response around the prosthesis in the control group, with synovial hyperplasia and infiltration by a large number of inflammatory cells. In the chitosan group, the levels of inflammatory cell infiltration and synovial hyperplasia were lower than those in the control group (Fig. 5).

### Does CMC promote bone deposition?

**Fig. 6:**
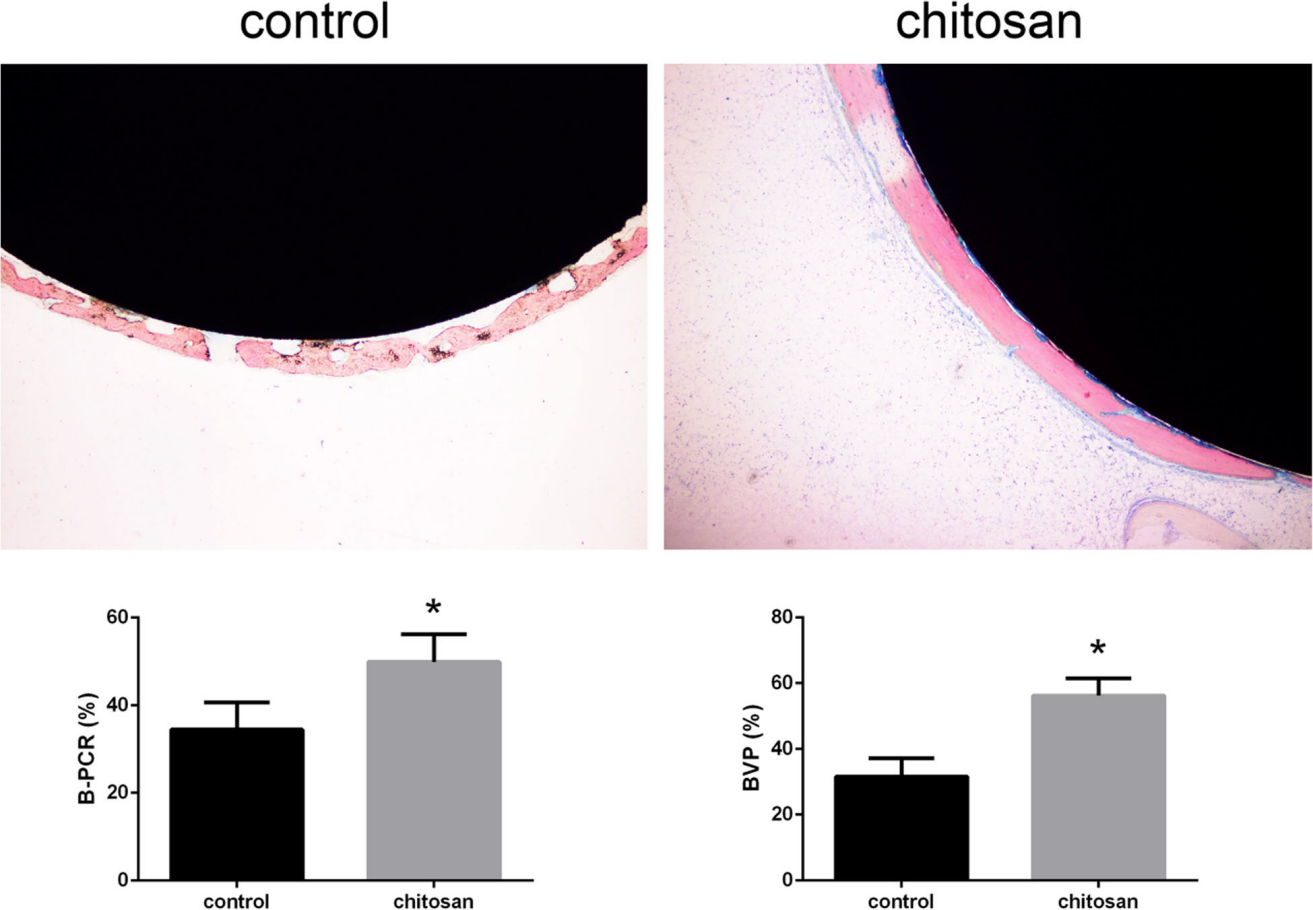
Van Gieson staining of bone tissue and hard tissue sections of the prosthesis (40x, bar = 250 μm). **a** The control group showed less bone formation around the prosthesis and intermittent bone formation(*n* = 10). **b** The chitosan group showed more bone formation around the prosthesis and continuous bone formation(*n* = 10). **c** The difference between the two groups’ bone-prosthesis contact rate and bone volume percentage was statistically significant (*p* < 0.05)

Hard tissue sections containing titanium rods received special staining. The VG staining results showed that there was more bone deposition in the chitosan group than in the control group, suggesting that the prosthesis was better in the chitosan group than in the control group. According to the staining results and the analysis of the image software IPP 6.0, The mean B-PCR value of the 10 samples in chitosan group was 49.872 ± 6.3350SD, and of the 10 samples in control group was 34.4570 ± 6.2216SD. Mean value of Trabecular number in chitosan group was 56.3050 ± 5.1722SD, and in control group was 31.633 ± 5.5007SD.B-PCR and BVP were significantly higher in the chitosan group than in the control group (*P* < 0.05) (Fig. 6).

**Fig. 7:**
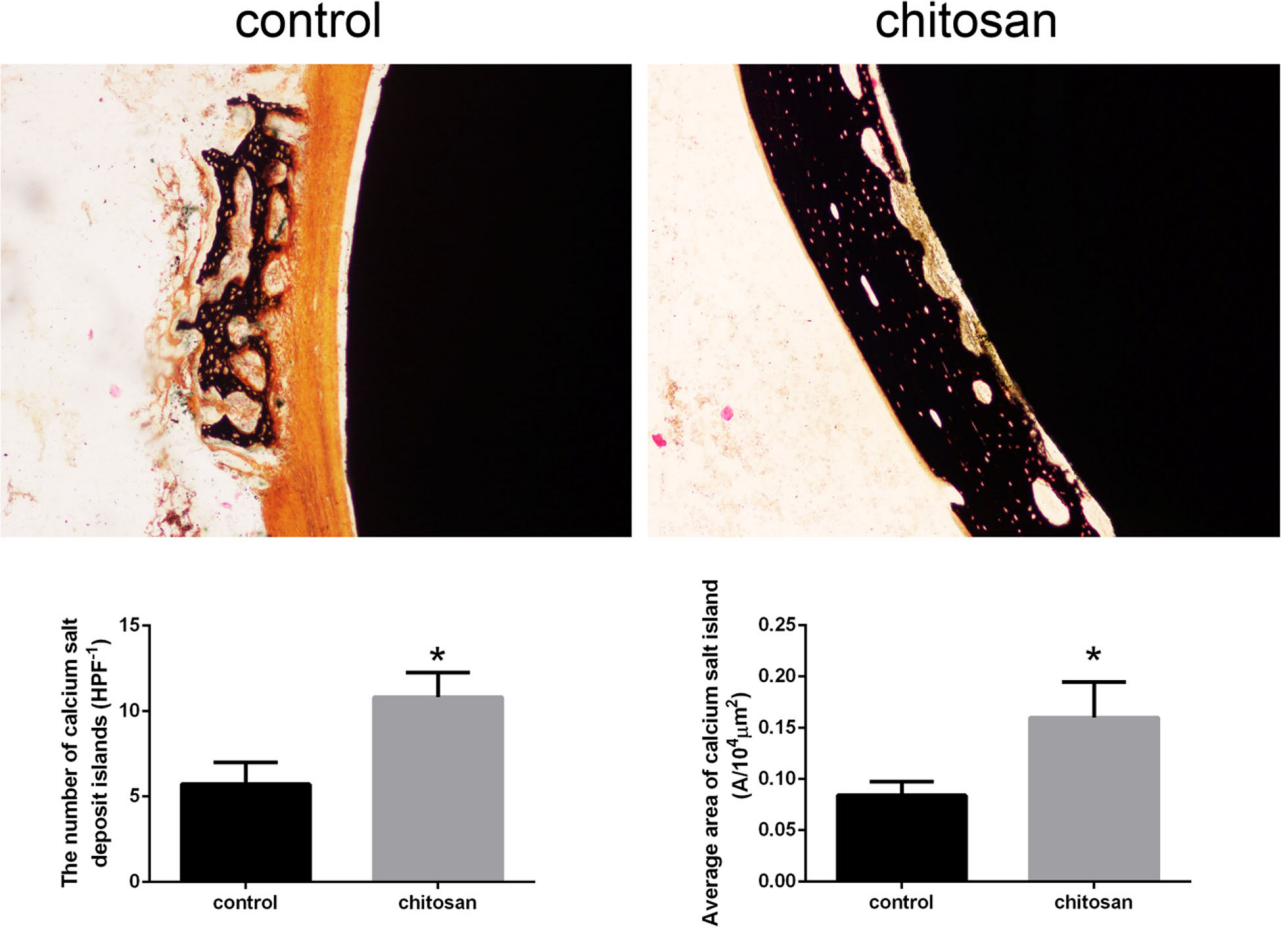
Von Kossa staining of bone tissue and hard tissue sections of the prosthesis (100x, bar = 100 μm). **a** Bone formation around the prosthesis in the control group was reticulated(*n* = 10). **b** Bone formation in the chitosan group was continuous(*n* = 10). **c** There was a statistically significant difference in the amount and area of calcium salt deposition between the two groups (*p* < 0.05)

Based on the Von Kossa staining results, we used IPP 6.0 to calculate CSDIs around the prosthesis in both groups. The CSDIs in the control group were mainly composed of a small amount of stellate. In contrast, the CSDIs in the chitosan group were linear and abundant. Mean value of the number of CSDIs in chitosan group was 10.801 ± 1.444SD, and in control group was 5.724 ± 1.267SD. The mean value of average area of CSDI in chitosan group was 0.16 ± 0.0346SD, and in control group was 0.084 ± 0.0135SD. The comparison between the two groups showed that the number and average area of CSDIs in the chitosan group were larger than those in the control group, and the difference was statistically significant (*P* < 0.05). The results showed that the control group was significantly worse than the chitosan group in terms of bone mineralization and deposition (Fig. 7).

### Effects of CMC on the metabolism of osteoblasts and osteoclasts

**Fig. 8:**
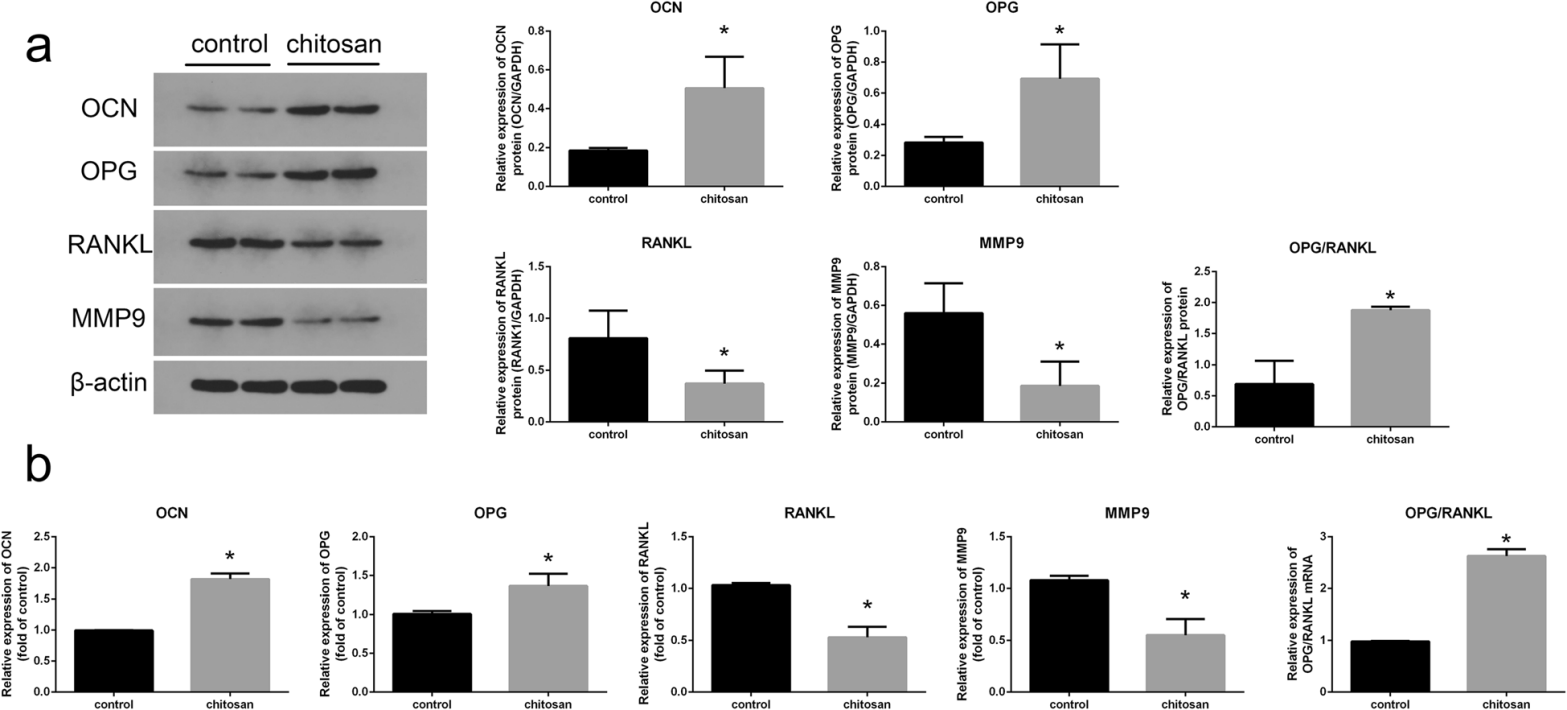
The expression changes of several key proteins (**a**) and genes (**b**) under chitosan treatment(*n* = 20). Compared with the control group, the expression of osteocalcin (OCN) and osteoprotegerin (OPG) in the chitosan group significantly increased (*p* < 0.05). In contrast, the expression of the receptor activator of nuclear factor kappa-Β ligand (RANKL) and matrix metalloproteinase-9 precursor (MMP9) in the chitosan group significantly decreased. The OPG/RANKL ratio significantly increased in the chitosan group (*p* < 0.05)

RT-PCR and WB were used to study specific mRNAs and proteins in the periprosthetic tissue. Two groups of specimens were processed simultaneously. The appropriate exposure time and developer based on β-actin were chosen. Compared with that of the control group, the osteoblast-specific marker OCN protein content of the chitosan group was significantly increased (*P* < 0.05), while the osteoclast-specific marker MMP9 protein content was decreased. Simultaneously, the content of OPG protein significantly increased, while the content of RANKL protein decreased in the chitosan group, resulting in a significantly increased OPG/RANKL ratio (P < 0.05). The OPG/RANKL balance plays an important role in maintaining bone metabolism. The increase in the OPG/RANKL ratio enhanced the activity of osteoblasts, weakened the activity of osteoclasts, promoted osteogenesis, and suppressed osteolysis. Considering the above results, we conclude that CMC around the titanium rod can promote bone formation and inhibit osteolysis (Fig. 8).

## Discussion

Aseptic loosening is one of the most common complications of surgical joint replacement [22]. It is a chronic and continuous pathological process accompanied by infiltration of inflammatory cells [17]. The inflammatory factors secreted by inflammatory cells activate the OPG/RANKL/RANK signalling pathway and promote osteolysis, eventually leading to aseptic loosening [23]. Therefore, it is possible to intervene in the loosening of the prosthesis through anti-inflammatory and anti-osteoporosis treatment.

Our experiments demonstrated that CMC has a positive effect on osteogenesis around prostheses. In this experiment, non-decalcified hard tissue sections were obtained from the prosthesis and its surrounding femur, and subsequently, special staining was performed. It demonstrated the actual condition of the contact between the prosthesis and bone tissue. The positive effects of CMC on osteogenesis and its beneficial anti-inflammatory effects were confirmed. Although there was a relatively slight difference in osteogenesis between the two groups during X-ray examination, micro-CT and VG staining of hard tissue sections showed that the BV/TV, B-PCR, and BVP levels of the chitosan group were higher than those of the control group. Von Kossa staining showed that the average area and number of CSDIs in the chitosan group were significantly higher than those in the control group, and most of them were continuous. The CSDIs in the control group were mostly networked and intermittent. These results suggest that the osteogenesis of the chitosan group is significantly better than that of the control group. We detected the synovial inflammatory response by HE staining, and there were significantly fewer inflammatory cells in the chitosan group than in the control group.

There are four advantages of CMC applied around the knee prosthesis. First, chitosan and its various compounds are widely used due to their biodegradability and biocompatibility, which does not affect the implantation of joint prostheses, can degrade itself and will not cause damage to surrounding tissues [24, 25]. Second, CMC has a broad-spectrum antibacterial effect, which interferes with the synthesis and metabolism of the bacterial outer wall, thereby causing damage to the structure of bacteria and inhibiting bacterial growth. At the same time, CMC reduces local inflammation by increasing lysozyme activity in macrophage responses and increases the body’s immune function [26]. Third, the biocompatibility of CMC makes bone cells easy to grow. Klokkevold et al. studied the differentiation of osteoblasts and bone formation in vitro, and the experimental results showed that chitosan can enhance the differentiation of bone progenitor cells and promote osteogenesis [27]. Finally, multiple studies have proven that chitosan and its derivatives have various antioxidant properties [28]. Yang et al. placed wear particles obtained from the interface membrane around a patient’s sterile loosening prosthesis into the primary osteoblasts of rabbits and found that the increased production of reactive oxygen species can induce osteoblast apoptosis [29]. The specific mechanism is that active oxygen mediates osteoblast apoptosis through the mitochondrial apoptotic protease-dependent pathway and the endoplasmic reticulum stress pathway, and this apoptosis can be attenuated by antioxidant antagonistic effects.

OPG/RANKL/RANK signalling is the most important pathway affecting osteogenesis. RANKL binds to its membrane-bound receptor nuclear factor receptor activation factor RANK, which stimulates osteoclast nuclear differentiation and maturation [23]. The soluble protein OPG is mainly produced in osteoblasts and acts as a RANKL-soluble bait receptor to inhibit the effect of RANKL [30]. The imbalance between RANKL and OPG is the key to osteoclast differentiation, osteolysis, and loss of bone mass. A review by Kobayashi et al. noted that the oxidative stress response can be accompanied by an increase in the expression of RANKL, while the OPG/RNAKL ratio decreases [31]. This means that oxidative stress can stimulate the RANKL/RANK interaction and inhibit the OPG/RANKL/RANK signalling pathway. The study of Xiao-Feng He et al. found that oxidative stress can induce osteoporosis through OPG/RANKL/RANK, and antioxidants can alleviate this bone loss [32]. The WB and real-time PCR results of the chitosan group in this study showed that the OPG/RANKL ratio increased, the expression of osteoblast-specific marker OCN increased, and the expression of osteoclast-specific marker MMP9 decreased, suggesting that CMC could promote bone formation by interfering with this signal.

However, our study has some limitations. First, this is not an exact TKA model, because the arthroplasty in the model was performed only for the femoral side. Second, the titanium rods used in this study were different from clinical prostheses, and they could not simulate human biomechanical mechanisms. Third, due to the irregular bone formation around the prosthesis, it was difficult to choose a fixed position to slice. We can only choose the slice with the thickest osteogenesis as a representative in the histological analysis. Finally, the experimental period was short, and the metabolism of CMC in each stage was not investigated. Hence, better TKA models and further large-scale studies are required in the future.

## Conclusion

This study investigated the effects of CMC in a TKA model. It has been preliminarily shown that CMC can reduce the inflammatory response around rabbit knee prostheses, affect the OPG/RANKL/RANK signalling pathway, and promote osteogenesis. This is of significant importance in preventing aseptic loosening of the knee joint prosthesis.

## Data Availability

The datasets used and analyzed during the current study are available from the corresponding author on reasonable request.

## Abbreviations

TKA: Total knee arthroplasty
CMC: Carboxymethyl chitosan
OPG: Osteoprotegerin
RANKL: Receptor activator of nuclear factor kappa-Β ligand
RNA: Ribonucleic acid
RT-PCR: Reverse transcription polymerase chain reaction
WB: Western blotting
CT: Computed tomography
BV/TV: Bone tissue volume/total volume of bone tissue and prosthesis
HE: Haematoxylin and eosin
VG: Van Gieson
B-PCR: Bone-prosthesis contact rate
BVP: Bone volume percentage
CSDIs: Calcium salt deposit islands
BCA: Bicinchoninic acid
SDS-PAGE: Sodium dodecyl sulfate polyacrylamide gel electrophoresis
MMP9: Matrix metalloproteinase-9 precursor
OCN: Osteocalcin
HRP: Horseradish peroxidase
ECL: Electrochemiluminescence
mRNA: Messenger RNA
IPP: Image-Pro Plus

## References

[1] Lohmander LS, Roos EM (2007). Clinical update: treating osteoarthritis. Lancet (London, England).

[2] Cram P, Lu X, Kates SL, Singh JA, Li Y, Wolf BR (2012). Total knee arthroplasty volume, utilization, and outcomes among Medicare beneficiaries, 1991-2010. Jama.

[3] Greidanus NV, Peterson RC, Masri BA, Garbuz DS (2011). Quality of life outcomes in revision versus primary total knee arthroplasty. J Arthroplast.

[4] Postler A, Lutzner C, Beyer F, Tille E, Lutzner J (2018). Analysis of Total knee Arthroplasty revision causes. BMC Musculoskelet Disord.

[5] Fehring TK, Odum S, Griffin WL, Mason JB, Nadaud M (2001). Early failures in total knee arthroplasty. Clin Orthop Relat Res.

[6] Mortazavi SM, Schwartzenberger J, Austin MS, Purtill JJ, Parvizi J (2010). Revision total knee arthroplasty infection: incidence and predictors. Clin Orthop Relat Res.

[7] Khan M, Osman K, Green G, Haddad FS (2016). The epidemiology of failure in total knee arthroplasty: avoiding your next revision. Bone Joint J.

[8] Gosheger G, Hardes J, Ahrens H, Streitburger A, Buerger H, Erren M, Gunsel A, Kemper FH, Winkelmann W, Von Eiff C (2004). Silver-coated megaendoprostheses in a rabbit model--an analysis of the infection rate and toxicological side effects. Biomaterials.

[9] Qin CQ, Huang DS, Zhang C, Song B, Huang JB, Ding Y (2016). Lentivirus-mediated short hairpin RNA interference targeting TNF-alpha in macrophages inhibits particle-induced inflammation and osteolysis in vitro and in vivo. BMC Musculoskelet Disord.

[10] Ulrich-Vinther M, Carmody EE, Goater JJ, Sb K, O'Keefe RJ, Schwarz EM (2002). Recombinant adeno-associated virus-mediated osteoprotegerin gene therapy inhibits wear debris-induced osteolysis. J Bone Joint Surg Am.

[11] Younes I, Rinaudo M (2015). Chitin and chitosan preparation from marine sources. Structure, properties and applications. Marine Drugs.

[12] LogithKumar R, KeshavNarayan A, Dhivya S, Chawla A, Saravanan S, Selvamurugan N (2016). A review of chitosan and its derivatives in bone tissue engineering. Carbohydr Polym.

[13] Fiamingo A, Campana-Filho SP (2016). Structure, morphology and properties of genipin-crosslinked carboxymethylchitosan porous membranes. Carbohydr Polym.

[14] Huang Y, Huang J, Cai J, Lin W, Lin Q, Wu F, Luo J (2015). Carboxymethyl chitosan/clay nanocomposites and their copper complexes: fabrication and property. Carbohydr Polym.

[15] Kong M, Chen XG, Xing K, Park HJ (2010). Antimicrobial properties of chitosan and mode of action: a state of the art review. Int J Food Microbiol.

[16] Ramasamy P, Subhapradha N, Thinesh T, Selvin J, Selvan KM, Shanmugam V, Shanmugam A (2017). Characterization of bioactive chitosan and sulfated chitosan from Doryteuthis singhalensis (Ortmann, 1891). Int J Biol Macromol.

[17] Xu H, Guo CC, Gao ZY, Wang CY, Zhang HN. Micrometer-Sized Titanium Particles Induce Aseptic Loosening in Rabbit Knee. Biomed Res Int. 2018;2018:5410875. 10.1155/2018/5410875 PMC5831897 29651439

[18] Lin HH, Peng SL, Wu J, Shih TY, Chuang KS, Shih CT (2017). A novel two-compartment model for calculating bone volume fractions and bone mineral densities from computed tomography images. IEEE Trans Med Imaging.

[19] Wedemeyer C, Neuerburg C, Pfeiffer A, Heckelei A, Bylski D, von Knoch F, Schinke T, Hilken G, Gosheger G, von Knoch M (2007). Polyethylene particle-induced bone resorption in alpha-calcitonin gene-related peptide-deficient mice. J Bone Mineral Res.

[20] Fujii J, Niida S, Yasunaga Y, Yamasaki A, Ochi M (2011). Wear debris stimulates bone-resorbing factor expression in the fibroblasts and osteoblasts. Hip Int.

[21] Maoqiang L, Zhenan Z, Fengxiang L, Gang W, Yuanqing M, Ming L, Xin Z, Tingting T (2010). Enhancement of osteoblast differentiation that is inhibited by titanium particles through inactivation of NFATc1 by VIVIT peptide. J Biomed Mater Res A.

[22] Lum ZC, Shieh AK, Dorr LD (2018). Why total knees fail-a modern perspective review. World J Orthopedics.

[23] Hofbauer LC, Kuhne CA, Viereck V (2004). The OPG/RANKL/RANK system in metabolic bone diseases. J Musculoskelet Neuronal Interact.

[24] Venkatesan J, Kim SK (2010). Chitosan composites for bone tissue engineering--an overview. Marine Drugs.

[25] Deepthi S, Venkatesan J, Kim SK, Bumgardner JD, Jayakumar R (2016). An overview of chitin or chitosan/nano ceramic composite scaffolds for bone tissue engineering. Int J Biological Macromolecules.

[26] Sahariah P, Masson M. Antimicrobial Chitosan and Chitosan Derivatives: A Review of the Structure-Activity Relationship. Biomacromolecules. 2017;18(11):3846–68. 10.1021/acs.biomac.7b01058 28933147

[27] Klokkevold PR, Vandemark L, Kenney EB, Bernard GW (1996). Osteogenesis enhanced by chitosan (poly-N-acetyl glucosaminoglycan) in vitro. J Periodontol.

[28] Chang SH, Wu CH, Tsai GJ (2018). Effects of chitosan molecular weight on its antioxidant and antimutagenic properties. Carbohydr Polym.

[29] Yang F, Tang J, Dai K, Huang Y (2019). Metallic wear debris collected from patients induces apoptosis in rat primary osteoblasts via reactive oxygen speciesmediated mitochondrial dysfunction and endoplasmic reticulum stress. Mol Med Rep.

[30] Rochette L, Meloux A, Rigal E, Zeller M, Malka G, Cottin Y, Vergely C (2019). The role of Osteoprotegerin in vascular calcification and bone metabolism: the basis for developing new therapeutics. Calcif Tissue Int.

[31] Kobayashi Y, Udagawa N, Takahashi N (2009). Action of RANKL and OPG for osteoclastogenesis. Crit Rev Eukaryot Gene Expr.

[32] He XF, Zhang L, Zhang CH, Zhao CR, Li H, Zhang LF, Tian GF, Guo MF, Dai Z, Sui FG (2017). Berberine alleviates oxidative stress in rats with osteoporosis through receptor activator of NF-kB/receptor activator of NF-kB ligand/osteoprotegerin (RANK/RANKL/OPG) pathway. Bosnian J Basic Med Sci.

